# A SHIME^®^-Based *In Vitro* Study to Simulate Human Gut Microbiota Modulation by Polyphenols from Pomegranate Juice

**DOI:** 10.3390/antiox15081047

**Published:** 2026-08-21

**Authors:** Angelica Bruno, Massimo Ferrara, Vito Linsalata, Angela Cardinali, Isabella D’Antuono

**Affiliations:** Institute of Sciences of Food Production, National Research Council (CNR-ISPA), Via G. Amendola 122/O, 70126 Bari, Italy; angelicabruno@cnr.it (A.B.); vito.linsalata@cnr.it (V.L.); angela.cardinali@cnr.it (A.C.)

**Keywords:** colonic microbiota, dynamic *in vitro* digestion, SCFA, pomegranate juice, anthocyanins

## Abstract

Polyphenol-rich pomegranate juice (PJ) has attracted growing interest in its health-promoting properties. However, most studies have focused on purified extracts rather than the whole beverage. Here, we evaluated the effects of whole PJ on gut microbiota composition and functionality using the Simulator of the Human Intestinal Microbial Ecosystem (SHIME^®^). The PJ was characterized by a high polyphenol content (2.3 g/L) and largely dominated by anthocyanins (~98% of identified compounds), with ellagic acid derivatives and flavonols present at lower concentrations. Following 7 days of treatment in the SHIME^®^ model, PJ polyphenols exhibited a bioaccessibility of 20–30%, in agreement with data from the literature. PJ supplementation induced a time-dependent modulation of the gut microbiota, leading to enhanced microbial fermentation and increased SCFA production, particularly in the descending colon, where total SCFA levels surpassed baseline values and reached 100 mmol/L. Moreover, PJ promoted the enrichment of beneficial taxa, including *Akkermansia*, supporting the ability of the whole juice matrix to improve gut microbial composition and metabolic activity. Overall, these findings support the potential of whole pomegranate juice as a functional food capable of modulating gut microbiota composition and activity.

## 1. Introduction

The gut microbiota, comprising trillions of microorganisms inhabiting the gastrointestinal tract, is increasingly recognized as a functional “organ” essential for human health. Through its involvement in nutrient metabolism, immune regulation, maintenance of intestinal barrier integrity, and production of bioactive metabolites such as short-chain fatty acids (SCFAs), it exerts profound effects on host physiology [1,2]. Alterations in microbial composition (dysbiosis) have been linked to several chronic diseases, including metabolic, inflammatory, cardiovascular, and neurological disorders [3,4].

Diet is one of the primary drivers of gut microbiota composition and functionality. Plant-based dietary patterns, including the Mediterranean diet, are associated with increased microbial diversity and enhanced abundance of SCFA-producing microorganisms, supporting beneficial host–microbiota interactions [5]. The rapid responsiveness of the microbiota to dietary changes highlights the potential of food bioactives, such as polyphenols, to shape microbial ecology and metabolic activity [6].

The health-promoting effects of dietary polyphenols largely depend on their bioaccessibility and bioavailability [7,8]. Following ingestion, polyphenols must be released from the food matrix during gastrointestinal digestion before becoming available for absorption and microbial metabolism [9]. Since these compounds are often embedded within complex plant matrices rich in dietary fibers and other macromolecules, their accessibility can be significantly influenced by both food characteristics and host-related factors [10,11]. Among these, the gut microbiota plays a pivotal role by converting polyphenols into a variety of metabolites with distinct biological activities and absorption profiles [12]. Therefore, assessing polyphenol bioaccessibility and bioavailability is essential for understanding the mechanisms through which polyphenol-rich foods modulate gut microbial communities and contribute to host health.

Pomegranate (*Punica granatum* L.), a hallmark Mediterranean fruit, is one of the richest natural sources of polyphenols, particularly anthocyanins, flavonols, and ellagitannins such as punicalagin [13]. Pomegranate polyphenols contribute to human health not only through their antioxidant and anti-inflammatory activities but also by modulating gut microbiota composition and functionality [14,15]. Preclinical studies have demonstrated that pomegranate extract supports the growth of beneficial bacteria while limiting pathogenic taxa, effects that are associated with improved metabolic and inflammatory outcomes [15,16,17]. However, the biological activity of these compounds is strongly influenced by their stability, bioaccessibility, and microbial biotransformation within the gastrointestinal tract, making the prediction of their in vivo effects challenging [18,19]. Therefore, a deeper understanding of the interactions between pomegranate polyphenols, host physiology, and microbial ecology is essential for the development of personalized nutritional strategies and for optimizing the therapeutic potential of pomegranate-derived products.

Dynamic *in vitro* digestion models coupled with gut microbiota simulators, such as the Simulator of the Human Intestinal Microbial Ecosystem (SHIME^®^), are increasingly used to investigate the gastrointestinal fate of dietary bioactives under controlled and reproducible conditions. By reproducing the main compartments of the human gastrointestinal tract and hosting a stabilized human microbiota, the SHIME^®^ model enables the simultaneous evaluation of polyphenol bioaccessibility, microbial metabolism, and microbiota responses [20]. Previous studies have explored the digestion and microbial transformation of pomegranate polyphenols, highlighting the conversion of ellagitannins into bioactive metabolites such as urolithins and demonstrating the key role of interindividual microbiota variability in these processes [21]. Furthermore, *in vitro* digestion and fermentation models have shown that gastrointestinal and microbial metabolism can enhance the availability of pomegranate phenolics and differentially affect microbial activity according to the composition and health status of the gut microbiota [22]. Nevertheless, important gaps remain. Most available studies have focused on isolated extracts or pomegranate-derived by-products, whereas the gastrointestinal fate of polyphenols within whole juice matrices and their effects on microbial communities throughout different colon regions remain poorly characterized.

Therefore, this study aimed to investigate the gastrointestinal fate of polyphenols from a whole-food matrix, namely pomegranate juice, using a dynamic SHIME^®^ model. By integrating polyphenol characterization, bioaccessibility assessment, SCFA production, and gut microbiota profiling, the study sought to provide a comprehensive evaluation of how the juice matrix influences polyphenol availability and gut microbiota responses along the gastrointestinal tract.

## 2. Materials and Methods

### 2.1. Chemicals

Ascorbic acid, Trolox, 1,1-Diphenyl-2-picryl-hydrazyl (DPPH), 2,2′-azinobis (3-ethylbenzothiazoline-6-sulfonic acid) diammonium salt (ABTS), Potassium persulfate, 2,4,6-Tris (2-pyridyl)-s-triazine (TPTZ), Ferric Chloride hexahydrate, Sodium Acetate, Folin–Ciocalteu reagent, aluminum chloride, sulphuric acid (H_2_SO_4_), methanol (MeOH), glacial acetic acid (AcOH) and ethyl acetate (EtOAc) were purchased from Sigma-Aldrich (Milan, Italy). The enzymes used for *in vitro* digestion (α-amylase from Bacillus sp., pepsin, pancreatin, mucin, lipase from pig; bovine bile extract) were obtained from Sigma-Aldrich (Milan, Italy). The polyphenol standards used in this study, such as cyanidin 3-glucoside chloride, cyanidin 3,5-glucoside chloride, delphinidin 3-glucoside chloride, delphinidin 3,5-glucoside chloride, ellagic acid, caffeic acid, quercetin, gallic acid and catechin, were purchased from PhytoLab GmbH & Co. KG (Vestenbergsgreuth, Germany). The SCFA external standards (acetic, propionic, butyric, valeric, caproic, isobutyric and isovaleric acids) were purchased from Sigma-Aldrich (Milan, Italy).

### 2.2. Sample Preparation and Determination of Polyphenolic Content and Antioxidant Activity

The pomegranate fruits were provided by the Apulian company Masseria Fruttirossi s.r.l. (Castellaneta, TA, Italy). The fruits belong to the Wonderful cultivar, characterized by large-sized fruit, a bright red color both outside and inside (arils), a balanced flavor with herbaceous sensory notes, a ripening season from October to March, harvesting taking place in November 2024. The pomegranate fruits were weighed, and the arils (edible part) were manually recovered, separating them from the internal and external peels. The arils were then manually squeezed to obtain the PJ, the seeds were removed, and the juice was filtered. Total phenolic content (TPC) was determined spectrophotometrically using the Folin–Ciocalteu method, as described by Singleton and Rossi, and results were expressed as milligrams of gallic acid equivalents per Liter of juice (mg GAE/L) [23]. Total flavonoid content (TFC) was determined using the aluminum chloride colorimetric method according to Barros et al. [24] and expressed as milligrams of catechin equivalents per Liter of juice (mg CE/L). Total anthocyanin content (TAC) was determined using the pH differential method as described by Lee et al. [25]. TAC was calculated using the following equation and expressed as milligrams of cyanidin-3-glucoside equivalents per Liter of juice (mg C3GE/L):

Anthocyanin pigment (mg C3GE/L) = (A × MW × DF × 10^3^)/(ε × l), (1)

where A is the absorbance difference [(A_510_ − A_700_) at pH 1.0 − (A_510_ − A_700_) at pH 4.5]; MW is the molecular weight of cyanidin-3-glucoside (449.2 g/mol); DF is the dilution factor; 10^3^ is the conversion factor from grams to milligrams; ε is the molar absorptivity of cyanidin-3-glucoside (26,900 L/mol·cm); and l is the optical path length (1 cm). Antioxidant activity was determined by DPPH, ABTS, and FRAP assays according to the method described by Iglesias-Carres et al. [26]. DPPH radical scavenging capacity was expressed as the sample concentration that give the 50% of DPPH inhibition (IC50) and as μmol TE/g DW, while the ABTS radical scavenging activity and the ferric reducing antioxidant power (FRAP) were expressed as μmol TE/g DW. The polyphenol concentration tested ranged from 25 to 100 μg/mL.

### 2.3. Polyphenolic Characterization and Quantification of Pomegranate Juice by HPLC DAD Analysis

The qualitative and quantitative characterization of polyphenols present in PJ was performed by HPLC-DAD analysis, using an Agilent 1260 Infinity system equipped with a 1260 binary pump, Hip Degasser, TCC thermostat, Diode Array Detector, and Agilent OpenLab Chem Station Rev. C 01.05 software (Agilent Technologies, Santa Clara, CA, USA). Chromatographic separation was achieved by using the Luna C18 reversed-phase column (4.6 × 250 mm, 5 μm particle size, Phenomenex, Torrance, CA, USA). The mobile phase consisted of methanol (eluent A) and water–acetic acid (95:5, *v*/*v*; eluent B), and the gradient program was applied according to previously published methods [27]. Chromatograms were monitored at two specific wavelengths to detect different phenolic classes, 360 nm (flavonoids) and 530 nm (anthocyanins). Compound identification was based on a comparison of retention times and UV-Vis spectra with those of available pure standards. In particular, delphinidin 3,5-diglucoside was quantified as delphinidin 3-glucoside, cyanidin glycoside as cyanidin 3-glucoside, ellagic acid derivative as ellagic acid and myricetin 3-O-glucoside as quercetin 3-glycoside. Results were expressed as micrograms of each compound per ml of juice (µg/mL).

### 2.4. In Vitro Digestion by Simulator of Human Intestinal Microbial Ecosystem (SHIME^®^)

The simulation of gastrointestinal and colonic digestion was performed using the Simulator of the Human Intestinal Microbial Ecosystem (SHIME^®^, Prodigest, Ghent, Belgium). In this study the TWIN-SHIME system was used, driving two replicates in parallel. The SHIME system replicates the stomach, the small intestine (SI), and the three regions of the colon, the ascending (AC), transverse (TC), and descending (DC) colon, which are connected through peristaltic pumps that enable fill-and-draw transfers between compartments. Additionally, the system provides automated control of temperature and pH, and the addition of media and compartment-specific enzymes. The procedure began with a stabilization phase, during which the intestinal microbiota was inoculated and maintained using a growth medium [20].

The TWIN-SHIME system was inoculated with a fresh fecal sample according to the SHIME^®^ manufacturer’s protocol (Prodigest, Ghent, Belgium). Briefly, the fecal inoculum was collected from a 45-year-old healthy human donor, living in Castellana Grotte (40.8874° N, 17.1663° E), following an omnivorous diet and not assuming antibiotics in the four months prior to sample collection. After inoculation of the single simulated colonic tracts, the fecal microbiota was incubated for 14 days anaerobically at 37 °C under continuous stirring condition at 200 rpm. This step establishes the baseline microbial population, allowing observation of microbial changes over time following the administration of PJ (dose of 5 mL/day) for 7 days [20]. Samples collected from the various compartments (SI, AC, TC, and DC) at different time points were analyzed chemically for bioaccessibility percentage and SCFA content and for microbiota profiling (AC, TC and DC only).

### 2.5. Bioaccessibility of Pomegranate Juice Polyphenols

HPLC-DAD analysis of digested samples collected from the SI reactor (*n* = 6), at the end of the digestive phase and prior to transfer to the first section of the colon (AC), was used to determine the bioaccessibility percentage of the major polyphenols identified in PJ, using the following formula:

Bioaccessibility% = CF/CI × 100,(2)

where CF represents the polyphenol content in the digesta, while CI represents the initial amount of polyphenols present in PJ, prior to digestion.

### 2.6. SCFA Extraction and Analysis by GC-FID

For SCFA extraction, 0.4 mL of colonic fermented samples was acidified with 80 μL of H_2_SO_4_ (50%), left at room temperature for 10 min and then for 1 h at 4 °C. Successively samples were mixed with 0.4 mL of ethyl acetate, vortexed for 5 min, incubated at 4 °C for 10 min and finally centrifuged at 13,000 g for 5 min [28]. Samples (1 μL of the organic phase) were analyzed in splitless mode by gas chromatography (Agilent 8890 GC Santa Clara, CA, USA, equipped with a flame ionization detector, GC-FID) using a DB-FATWAX Ultra Inert GC column (Agilent J&W, 30 m length, 0.25 mm ID, 0.25 μm film thickness) under the following conditions: initial temperature of the oven of 80 °C for 2.5 min, followed by heating to 235 °C at 15 °C/min and then to 245 °C at 30 °C/min, which was maintained for 2 min. The total run time was about 17 min, while the solvent delay on the detector was set at 4 min. The signal was detected at 250 °C with the FID detector. The GC injector was maintained at 250 °C and injection pulse pressure was set to 8.5 psi (3.0 mL/min), while the purge flow to the split vent was set to 50.0 mL/min at 3 min. After 0.55 min, the helium gas flow rate was set to constant flow rate of 1.5 mL/min (16.7 psi) [29]. The identification of SCFAs was confirmed using individual calibration curves of external standards (acetic, propionic, butyric, valeric, caproic, isobutyric and isovaleric acids). SigmaPlot 12.0 one-way ANOVA test, followed by the LSD’s post hoc test, was applied to establish significant differences between means (*p* < 0.05).

### 2.7. Colonic Microbiota Analysis

Metabarcoding analysis was performed to evaluate the modulation of the microbial composition upon PJ supplementation compared with the control. Samples were taken, in triplicates, from AC, TC, and DC SHIME^®^ colonic tracts after 0 h, 4 days and 7 days of PJ supplementation. The DNA was extracted from each pelleted microbiome, using the PureLink Microbiome DNA Purification Kit (Thermo Fisher Scientific, Waltham, MA, USA), according to the manufacturer’s recommendation. The DNA concentration (ng/μL) and purity (A260/A280 and A260/A230 ratio) were assessed using a NanoDrop OneC spectrophotometer (Thermo Fisher Scientific, Waltham, MA, USA). DNA integrity was evaluated by electrophoresis on 1.5% (*w*/*v*) agarose gel stained with GelRed (Biotium, Fremont, CA, USA). The V4 region of the 16SrRNA gene was amplified by using the universal primers 515F (5′-GTGCCAGCMGCCGCGGTAA-3′) and 806R (5′-GGACTACVSGGGTATCTAAT-3′). The amplification was performed using the Platinum SuperFi DNA Polymerase (Invitrogen, Carlsbad, CA, USA) applying the following conditions: 98 °C for 30 min, 25 cycles of 98 °C for 10 s, 58 °C for 20 s and 72 °C for 30 s and a final extension of 72 °C for 5 min. The libraries produced were verified, pooled and sequenced using the Ion Torrent platform (Ion GeneStudio S5 System, Thermo Fisher Scientific, Waltham, MA, USA), according to Gialluisi et al. [30]. The sequence data are available at NCBI SRA under BioProject ID: PRJNA1428230.

### 2.8. Bioinformatics

The raw reads were processed for quality checking and overall quality assessment using Qiime2 [31]. Primer sequences were trimmed using Cutadapt [32]. Denoising of reads was performed using qiime dada2 optimized for Ion Torrent sequences (HOMOPOLYMER_GAP_PENALTY = −1, BAND_SIZE = 32) [33]. The taxonomic classification of amplicon sequence variants (ASVs) was performed using the qiime feature-classifier classify-consensus-vsearch command against the reference SILVA database (v.138.1) database for taxonomic assignment. A rarefaction step at the lowest number of reads/samples was then performed to standardize the sequencing depth across samples. The alpha diversity index (including Shannon and Chao1) was calculated using the RAWGraphs 2.0 [34]. MicrobiomeAnalyst v2.0 2024-11-01 was used for Beta diversity analysis using principal coordinate analysis (PCoA) based on Bray–Curtis dissimilarity, with statistical significance tested via PERMANOVA and a significance threshold of an FDR-adjusted *p*-value  <  0.05 [35]. linear discriminant analysis (LDA) effect size (LEfSe) was performed to detect characteristic features with significant differential abundance in different categories [36]. Statistical comparisons were made using the Kruskal–Wallis and Wilcoxon tests, with an alpha value of 0.01. A cut-off threshold for the LDA (linear discriminant analysis) score was set at (log10) > 2.0.

## 3. Results and Discussion

### 3.1. Physicochemical and Antioxidant Characterization of Wonderful Pomegranate Juice

The production parameters, the polyphenolic composition, and the antioxidant activity of pomegranate fruit were reported in Table 1. Fruits showed an average weight of 554.4 ± 92.4 g and a juice yield of 33% (182.7 mL), values consistent with those previously reported for cv. Wonderful cultivated in Mediterranean regions [13]. The juice was characterized by a remarkably high total phenolic content (2329.8 ± 10.1 mg GAE/L), with anthocyanins as the most representative (602.6 ± 2.6 mg C3GE/L), in agreement with the typical intense red coloration of this cultivar [37,38]. The total flavonoid content reached 242.6 ± 3.1 mg CE/L, indicating the presence of additional bioactive phenolic subclasses. The antioxidant capacity of the juice was evaluated using complementary vitro assays. A low IC50 value in the DPPH radical scavenging assay (41.7 ± 3.3 mg/L) and high Trolox equivalent values measured by ABTS (13.3 ± 4.6 μmol TE/mL) and FRAP (19.7 ± 0.9 μmol TE/mL) assays demonstrated strong radical scavenging and reducing activities. These findings agreed with previous studies, which reported a close correlation between antioxidant activity and phenolic concentration, particularly anthocyanin and ellagitannin contents in PJ [39,40]. The strong antioxidant potential observed may be attributed not only to the concentration of phenolic compounds but also to potential synergistic interactions among anthocyanins, flavonoids, and phenolic acids, which may enhance their overall antioxidant activity.

### 3.2. Pomegranate Juice Polyphenol Composition and Their Bioaccessibility After In Vitro Digestion Process

**Figure 1:**
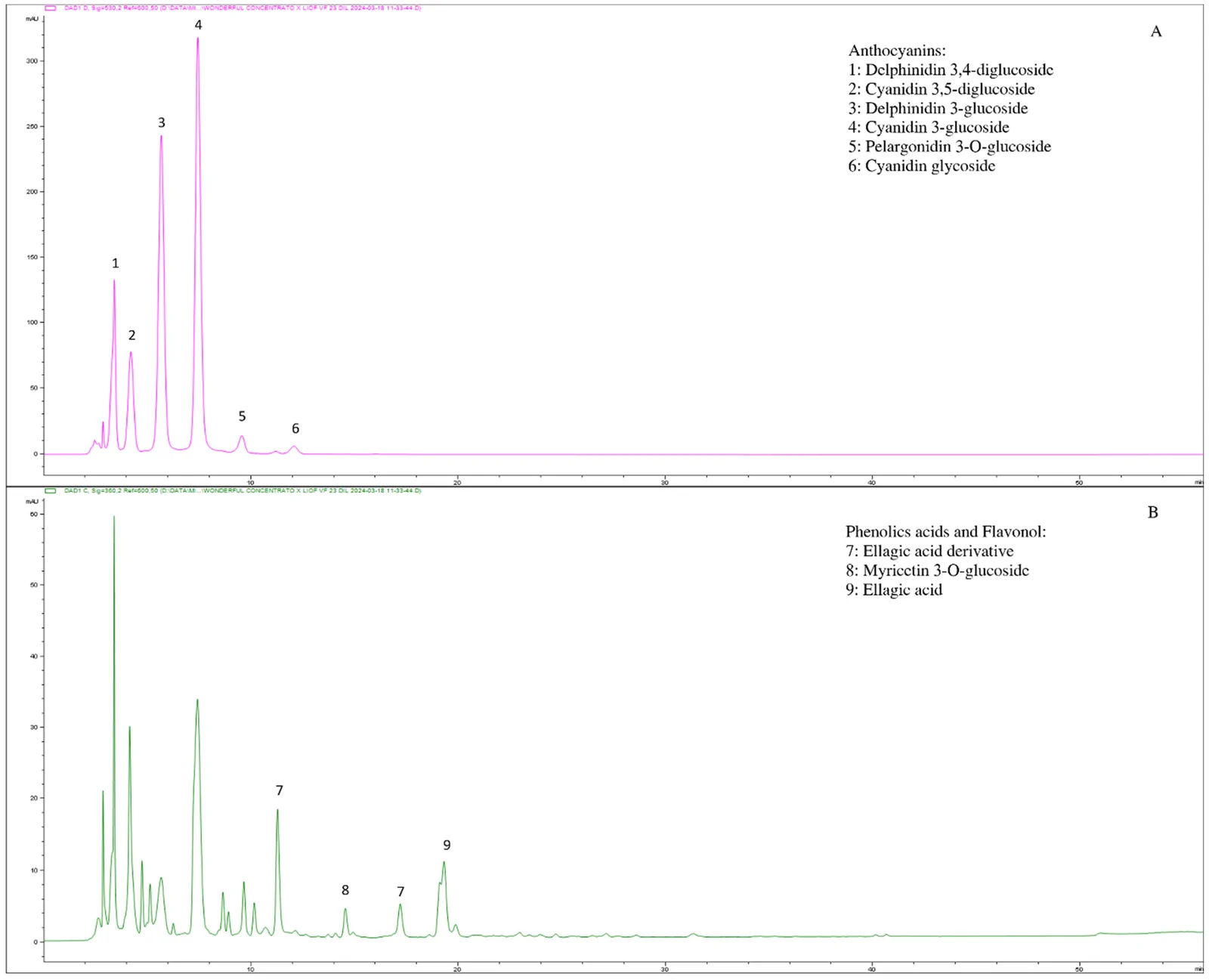
HPLC-DAD chromatograms of the identified polyphenols in pomegranate juice (PJ, cv. Wonderful), recorded at 520 nm (**A**) and 360 nm (**B**).

The polyphenolic profile of PJ, determined by HPLC-DAD analysis, is reported in Table 2, whereas chromatograms recorded at two different wavelengths (520 and 360 nm) are presented in Figure 1A and Figure 1B, respectively.

HPLC-DAD analysis confirmed that pomegranate juice (PJ) was characterized by a polyphenol-rich profile, with a total identified polyphenol concentration of approximately 2.3 g/L. Anthocyanins represented the predominant class, accounting for nearly 98% of the identified polyphenols (Table 2). The major compounds were cyanidin 3,5-diglucoside (C 3,5-diglu), delphinidin 3-O-glucoside (D 3-glu), cyanidin 3-O-glucoside (C 3-glu), and delphinidin 3,5-diglucoside (D 3,5-diglu), while pelargonidin 3-O-glucoside and cyanidin glycosides were detected in lower amounts. Minor amounts of pelargonidin 3-O-glucoside and cyanidin glycoside were also detected. This profile is consistent with previous reports identifying cyanidin and delphinidin diglucosides as characteristic components of pomegranate juice from different cultivars, including Wonderful [13]. The predominance of anthocyanins is likely related to the juice extraction procedure adopted in this study. As reported by Fischer et al. [38], juice extracted exclusively from the arils, as in the present study, exhibits a significantly higher anthocyanin content compared to juices obtained from pressing the whole fruit (including mesocarp and peel). This finding also explains the absence of ellagitannins in the juice, as they are predominantly concentrated in the peel. Besides anthocyanins, lower concentrations of ellagic acid (EA), ellagic acid derivatives, and myricetin 3-O-glucoside (M 3-glu) were detected. The presence of ellagic acid agrees with previous studies describing it as an important phenolic constituent and hydrolysis product of ellagitannins in pomegranate-derived products [40]. Although present at lower concentrations than anthocyanins, EA and its derivatives may substantially contribute to the overall antioxidant capacity of the juice through their potent free radical scavenging activity and complementary antioxidant mechanisms. In combination with anthocyanins, these compounds may contribute to the health-promoting properties of pomegranate juice, including anti-inflammatory and cardiovascular protective effects [14,40]. In addition, anthocyanins have been linked to metabolic benefits, including the modulation of intestinal glucose absorption. Indeed, Barberis et al. [41] demonstrated, using a Caco-2 TC7 cell model coupled with biosensors, that Wonderful pomegranate juice reduced glucose uptake compared with the control, highlighting the potential contribution of anthocyanins to its hypoglycemic activity.

**Figure 2:**
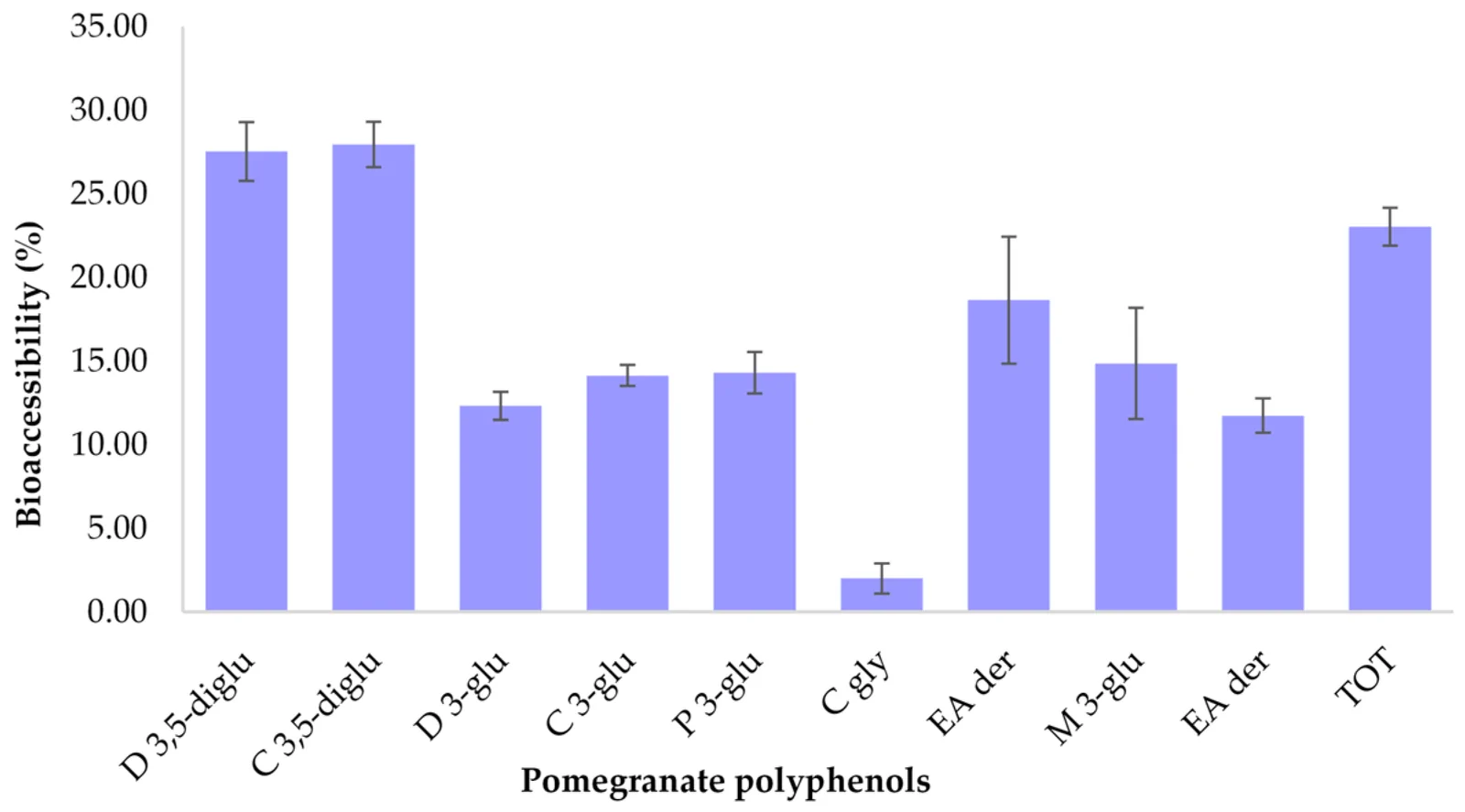
Bioaccessibility of the main pomegranate juice polyphenols (cv Wonderful): D 3,5-diglu, delphinidin 3,5-diglucoside; C 3,5-diglu, cyanidin 3,5-diglucoside; D 3-glu, delphinidin 3-O-glucoside; C 3-glu, cyanidin 3-O-glucoside; P 3-glu, pelargonidin 3-O-glucoside; C gly, cyanidin glycoside; EA der, ellagic acid derivative; M 3-glu, myricetin 3-O-glucoside; EA, ellagic acid; TOT, total polyphenols identified.

Figure 2 shows the bioaccessibility (%) of the main polyphenols in PJ following gastrointestinal digestion using the SHIME^®^ dynamic model. These data provide valuable insights into the behavior of individual polyphenolic compounds during digestion, which may ultimately influence their potential bioavailability and associated health effects. The gastrointestinal environment affects the stability and release of PJ polyphenols, resulting in an overall bioaccessibility of approximately 24% of the initial polyphenol content.

This finding is consistent with previous studies reporting bioaccessibility values ranging from 20% to 35% for total pomegranate polyphenols [18]. The results obtained are in agreement with the well-established evidence that anthocyanins are relatively stable under the acidic conditions of the stomach but undergo varying degrees of degradation in the alkaline environment of the duodenum [19]. Furthermore, the absence of a solid food matrix, as occurs in liquid products such as fruit juice, may reduce the polyphenol stability during digestion. Among the compounds analyzed, D 3,5-diglu and C 3,5-diglu showed the highest bioaccessibility values (~28–29%), suggesting that these diglucosylated anthocyanins are comparatively more stable throughout both the gastric and intestinal phases of digestion. This observation is consistent with previous studies highlighting the greater stability of anthocyanin diglucosides compared with their monoglucoside counterparts, as diglucosylation may confer protection against enzymatic hydrolysis and acidic conditions, thereby enhancing their persistence throughout the intestinal environment [42]. Ellagic acid and its derivatives exhibited moderate bioaccessibility (12–18%), likely due to their limited solubility and reduced stability under physiological digestive conditions. However, their potential microbial conversion into bioactive urolithins, as documented in previous studies, may substantially contribute to their biological relevance [43,44]. Overall, these findings emphasize the importance of considering not only the polyphenol content of foods but also the transformation they undergo, as well as their stability and bioaccessibility throughout the digestive process, when evaluating their potential health benefits.

### 3.3. Effect of Pomegranate Juice Intake on Gut Microbiota in the SHIME^®^ System

#### 3.3.1. SCFA Analysis

**Figure 3:**
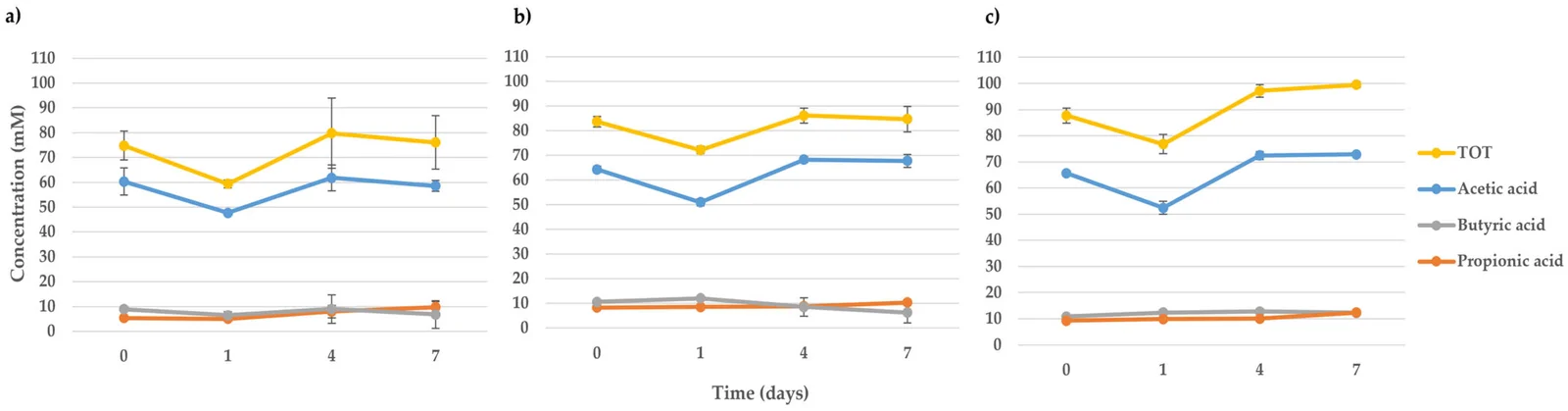
Millimolar concentration of major SCFAs in ascending (**a**), transverse (**b**) and descending (**c**) colon regions, at 0, 1, 4, 7 days of pomegranate juice administration. Data were presented as the means ± SD. Statistical differences are reported in Table S1.

The metabolic activity of colonic microbiota was assessed through quantitative analysis of SCFAs, and the results were reported in Figure 3 as mean values. The figure shows the millimolar concentration of SCFAs in each compartment of the colon, AC, TC and DC, at 0, 1, 4, 7 days of PJ administration to the system.

The most abundant SCFAs were acetic, propionic and butyric acids, which together accounted for more than 95% of the total SCFAs. Their approximate molar ratio was 70:15:15, in agreement with values reported in the literature [45,46]. Total SCFA concentration decreased after the first administration of PJ (day 1), likely as a consequence of an initial shift in the microbial community and its metabolic activity following juice supplementation. SCFA concentration subsequently increased on days 4 and 7, returning to baseline levels in the AC and TC compartments and exceeding the initial values in the DC. The highest total SCFA production was recorded in DC after 7 days of PJ administration, reaching a concentration of 100 mmol/L. In this compartment, the increase in total SCFAs as well as in acetic, propionic and butyric acids at day 7 were statistically different compared with day 0 (Table S1). In contrast, no significant differences were observed in the AC and TC compartments over the same period (Table S1). Concerning the minor components, SCFA, isobutyric acid and isovaleric acid concentrations were higher in the DC than in the AC and TC at day 7 and remained relatively stable thereafter, suggesting adaptation of the microbial community to PJ supplementation. Conversely, caproic and valeric acid concentration remained relatively constant throughout the experiment across all colonic compartments, ranging between 0.2 and 0.3 mM (Figure S1).

The increase in the production of these compounds is closely associated with changes in the gut microbial community. Among the three main SCFAs, butyrate is considered particularly important for colonic health, as it represents the primary energy source for colonocytes and exerts well-documented anti-inflammatory effects [45]. The consumption of oxygen for the oxidation of butyrate by colonocytes creates a hypoxic mucosal environment that inhibits the proliferation of facultative aerobic pathogens such as *Salmonella* spp. and *Escherichia coli*. Consequently, butyrate-producing bacteria play a key role in maintaining intestinal homeostasis. Among the most relevant taxa are *Roseburia* and *Faecalibacterium prausnitzii*, both belonging to the phylum Bacillota (formerly Firmicutes) [47]. Reduced abundance of these taxa has been consistently associated with various pathological conditions, particularly inflammatory bowel disease (IBD) [48].

Several studies have investigated the effects of pomegranate-derived products on SCFA production and gut microbiota modulation. Sivamani et al. [17] reported that pomegranate extract supplementation increased the abundance of SCFA-producing bacteria, including *Roseburia*, *Faecalibacterium prausnitzii*, and *Coprococcus eutectus*. Similarly, Bialonska et al. [15] demonstrated that pomegranate by-product extracts selectively promoted the growth of *Lactobacillus* and *Bifidobacterium* spp. in distal colon batch fermentations, accompanied by increased acetate, propionate, and butyrate production. Likewise, Li et al. observed that both pomegranate extract and juice stimulated the growth of *Bifidobacterium* and *Lactobacillus*, while reducing the abundance of potentially undesirable bacterial groups, including the *Bacteroides fragilis* group, *Clostridia* and *Enterobacteriaceae* [49]. Consistent with previous evidence, pomegranate juice administration in the present study was associated with increased SCFA production, suggesting an enhanced metabolic activity of beneficial gut microorganisms.

#### 3.3.2. Colonic Microbiota Analysis

**Figure 4:**
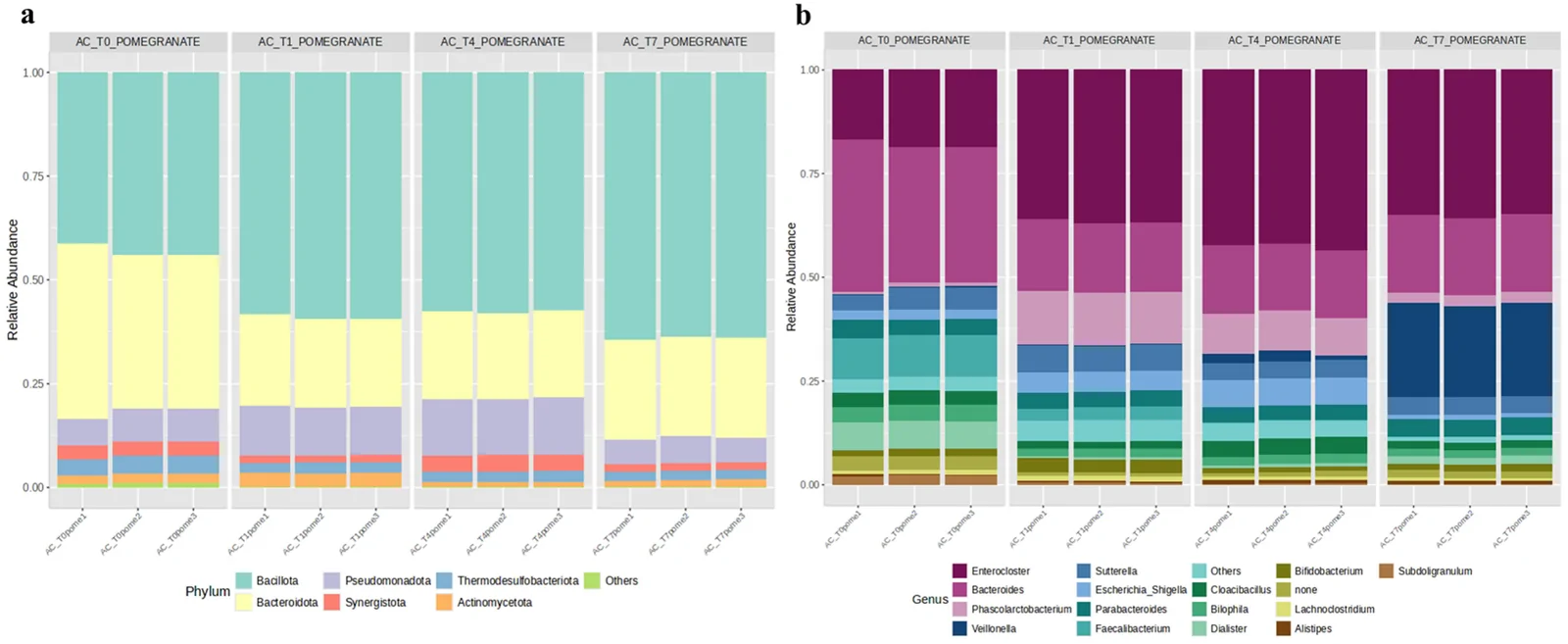
Relative abundance at phylum (**a**) and genus (**b**) levels of colonic microbiota in ascending colon segment (AC), supplemented with pomegranate juice.

Metagenomic analysis was performed to monitor changes in the colonic microbiota across the AC, TC, and DC compartments, from T0 to T7 in the SHIME^®^ system. The alpha diversity analysis revealed differences in gut microbiota diversity across the various time points. In the AC segment, the Shannon index decreased during pomegranate juice administration (Figure S2). This effect could be related to the potential inhibitory activity of polyphenols immediately after administration. Polyphenol-rich foods frequently exert selective antimicrobial activity against specific bacterial groups, leading to an initial reduction in community evenness while simultaneously promoting the expansion of taxa better adapted to metabolize phenolic compounds. In TC, alpha diversity increased after 1 day of administration and decreased on the fourth and seventh days of administration. For the DC segment, Shannon index revealed minor changes in alpha diversity, although not significant. The beta diversity plots indicate that the microbial community in AC is distinct at T0 from the other sampling time points, with T1, T4, and T7 showing a shift in community structure as the pomegranate treatment progresses (Figure S3). A similar trend was observed even in TC and DC with stronger evidence after 7 days of administration (Figures S4 and S5). At the taxonomic level, the AC compartment was dominated by Bacillota and Bacteroidota at the phylum level. From T1 to T7, a decrease in the relative abundance of Pseudomonadota and Actinomycetota was observed (Figure 4a). Additionally, an increase in the Bacillota/Bacteroidota ratio (formerly the F/B ratio) was observed in all three colon compartments, with this increase being more pronounced in the AC region. The increase in the Bacillota/Bacteroidota ratio observed throughout the SHIME^®^ compartments may reflect selective stimulation of bacterial groups involved in carbohydrate fermentation and SCFA production.

At T0, *Enterocloster* and *Bacteroides* were the primary genera in the AC compartment (Figure 4b). A notable decrease in *Bacteroides* and an increase in *Enterocloster* and *Veillonella* were detected from T0 to T7. *Bacteroides* are versatile primary degraders of complex polysaccharides. They play a fundamental role in the breakdown of plant-derived compounds that the human host cannot digest. The *Enterocloster* genus is involved in the fermentation of complex carbohydrates and the production of butyrate, a primary energy source for colonocytes. In the context of pomegranate metabolism, members of the aforementioned families are frequently involved in the biotransformation of ellagitannins and their derivatives into urolithins. In addition, microbial enzymes produced by various gut microbiota families are capable of deglycosylating polyphenols, particularly flavonoids and anthocyanins, with a specific action on O-glycosylated compounds [50]. Anthocyanins, which were the main polyphenols identified in our PJ, are known to exert prebiotic effects by increasing the abundance of beneficial bacteria in the gastrointestinal tract. These compounds promote the production of SCFAs and lactic acid in the intestine, thereby creating favorable conditions for the growth of probiotic microorganisms. Furthermore, anthocyanins can serve as a carbon source for probiotics and stimulate bacteriocin production, enhancing their competitive ability against pathogenic bacteria in the gut [51]. Moreover, the genus of *Veillonella* is known for metabolizing lactate (produced by other bacteria like *Bifidobacterium*) into propionate, an important SCFA for metabolic regulation.

**Figure 5:**
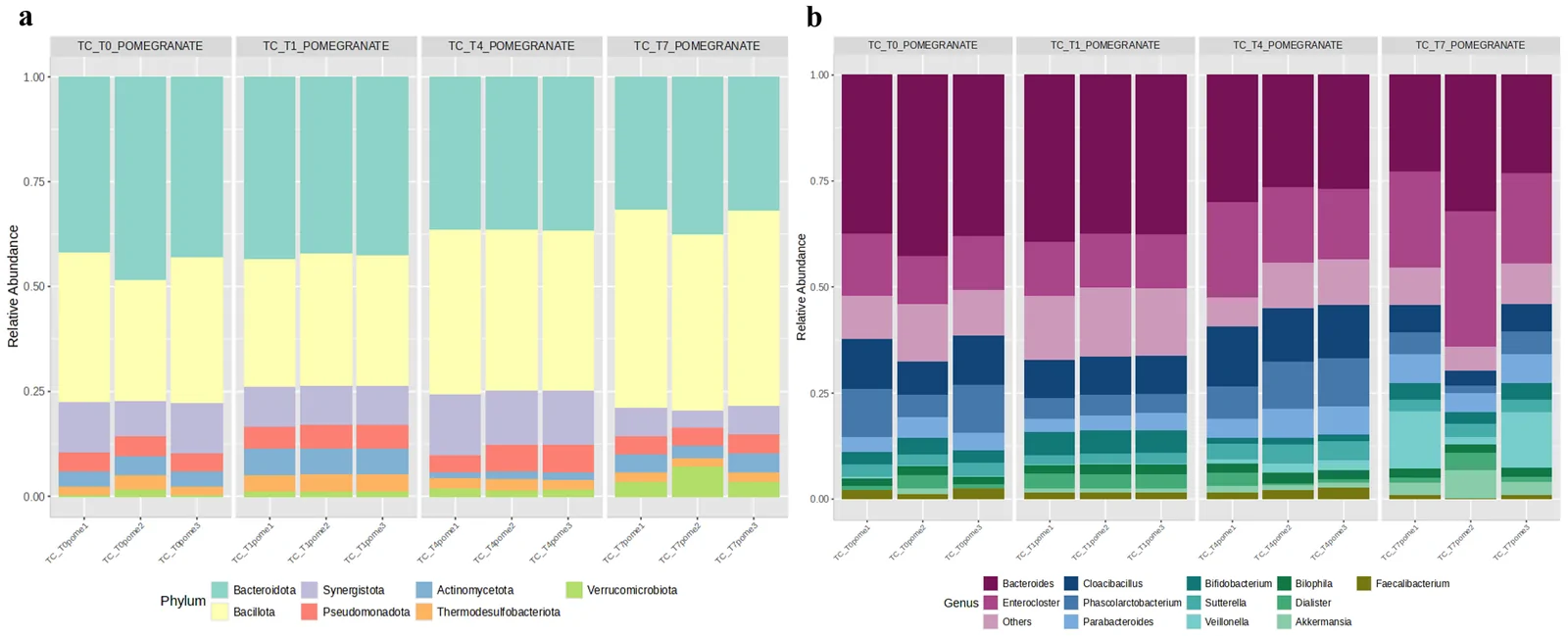
Relative abundance at phylum (**a**) and genus (**b**) levels of colonic microbiota in transverse colon segment (TC), supplemented with pomegranate juice.

**Figure 6:**
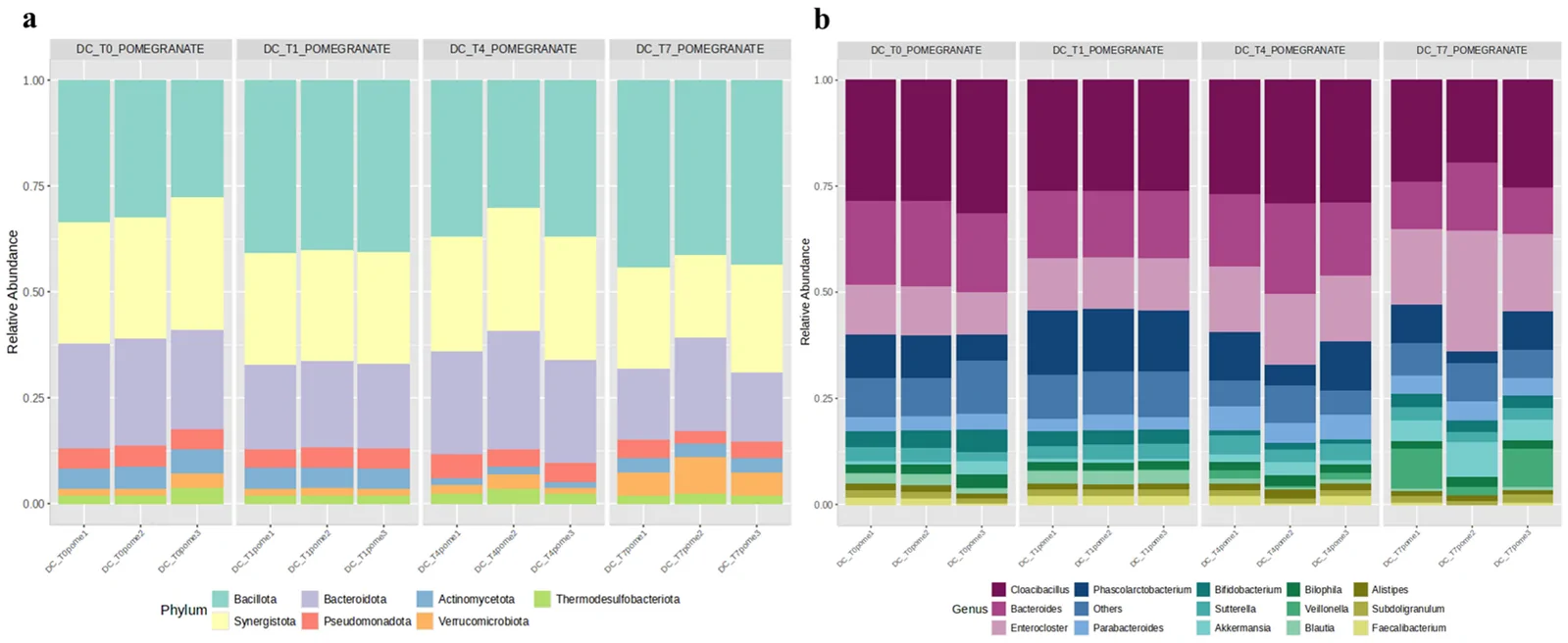
Relative abundance at phylum (**a**) and genus (**b**) levels of colonic microbiota in descending colon segment (DC), supplemented with pomegranate juice.

Similar to the AC, the TC shows a strong baseline of Bacteroidota and Bacillota phyla. A significant increase by T7 was observed for the Verrucomicrobiota phylum (Figure 5 and Figure 6).

At the genus level, the TC segment was dominated by *Bacteroides* and *Enterocloster*, although in different proportions with respect to AC. Moreover, *Akkermansia* shows a clear increase in relative abundance at T7 compared to T0. *Phascolarctobacterium*, *Parabacteroides* and *Bifidobacterium* also show consistent presence along the administration period.

In the DC colonic segment, a marked increase in Verrucomicrobiota phylum by T7 was observed (Figure 6).

The relative abundance of Bacteroidota appears to decrease slightly, while Synergistota became more pronounced in the DC region, respect to the AC and TC segments. At the genus level *Cloacibacillus*, *Bacterioides*, *Enterocloster* and *Phascolarctobacterium* were the most represented genera at all time point analysis. Interestingly the *Cloacibacillus* genus progressively increased its presence from the AC segment to the DC segment, being the most representative genus in the last segment of SHIME^®^ system. Notably, *Akkermansia* showed increased abundance at T7, when compared to T0.

The observed changes, particularly the increase in *Akkermansia* and, to a lesser extent, *Bifidobacterium*, are consistent with the known prebiotic effects of pomegranate polyphenols [21,51]. Although recent studies using the SHIME^®^ system and pomegranate extract did not report an increase in the genus *Akkermansia* [52,53], the increase observed in our study at T7 in the TC and DC compartments is consistent with the findings of Henning et al. [54], suggesting that pomegranate polyphenols may act as substrates that promote the growth of this mucin-degrading bacterium, which is associated with improved metabolic health. The genus *Akkermansia* includes mucin-degrading species that inhabit the protective mucus layer of the gut. It is widely regarded as a marker of gut health because it contributes to the maintenance of gut barrier integrity and exerts anti-inflammatory effects. Its increased abundance has frequently been associated with improved metabolic health and glucose homeostasis. Moreover, the observed increase in genera such as *Enterocloster* (formerly classified within the family *Lachnospiraceae*) during PJ administration is particularly relevant because certain members are involved in the microbial metabolism of ellagic acid (EA), leading to the formation of urolithin intermediates. This genus also participates in the fermentation of complex carbohydrates and the production of butyrate, a primary energy source for colonocytes. The SHIME^®^ system was particularly effective in demonstrating that the modulation of gut bacterial communities occurred predominantly in the TC and DC compartments, where they are extensively metabolized low-molecular-weight compounds, such as phenolic acids and urolithins, which are often more bioactive and bioavailable than the parent compounds [55]. The stability of the overall microbial load during these taxonomic shifts suggests that the pomegranate treatment shaped the microbial community toward a more beneficial profile. Nevertheless, considering the well-recognized interindividual variability of the gut microbiota, which is influenced by factors including age, diet, geographical location, and lifestyle, our findings could be confirmed by further studies, involving multiple donors, in order to assess their broader applicability.

**Figure 7:**
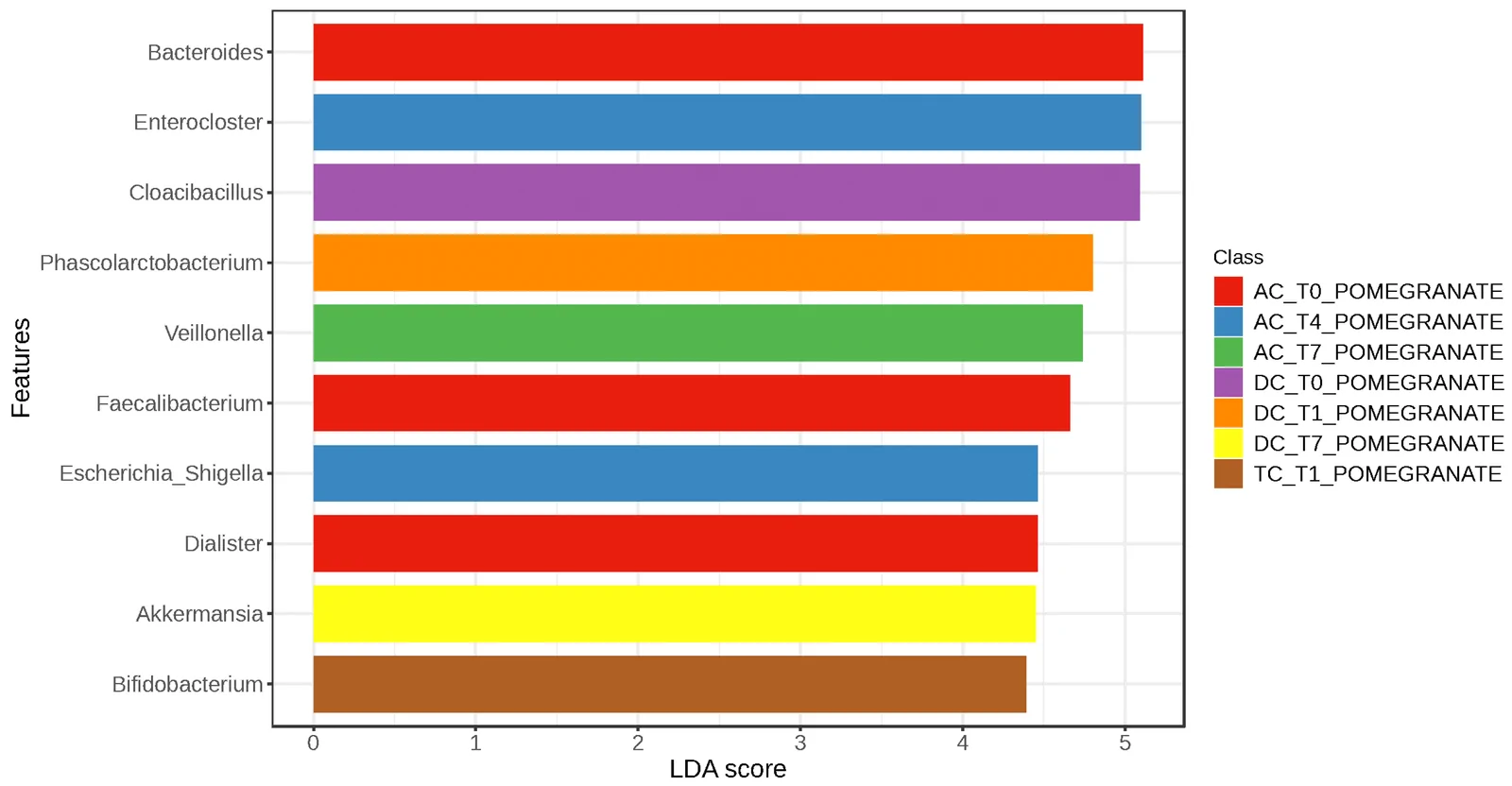
Histogram of the top 10 linear discriminant analysis (LDA) scores for differentially abundant genera combined with effect size measurements (LEfSe) with a list of genera (y-axis) that significantly changed in frequency after PJ administration, with respect to the different colonic segments. *p* < 0.05 was considered significant in Kruskal–Wallis and a score ≥ 2.0 was considered significant in pairwise Wilcoxon test. AC = ascending colon, TC = transverse colon, DC = descending colon.

LDA combined with LEfSe provided further evidence that PJ supplementation promoted a time-dependent ecological succession within the gut microbial community [35]. Specifically, among the 10 top scored genera (Figure 7), *Bateroides*, *Faecalibacterium* and *Dialister* were associated with AC at T0, while *Enterocloster*, *Escherichia* and *Shigella* were significantly associated at T4 and *Veillonella* at T7. For TC, *Bifidobacterium* was significantly associated with this colonic segment at T1, while in the DC segment *Cloacibacillus*, *Phascolarctobacterium*, and *Akkermansia* were significantly associated at T0, T1 and T7, respectively. The strong association of *Akkermansia* with the DC segment after 7 days of pomegranate administration highlighted the beneficial effect of pomegranate juice consumption in promoting gut microbiota health.

## 4. Conclusions

In conclusion, pomegranate juice (cv. Wonderful) proved to be a rich source of bioactive polyphenols, particularly anthocyanins, and demonstrated strong antioxidant activity. Although the overall bioaccessibility of polyphenols during simulated gastrointestinal digestion was approximately 25%, a substantial proportion, particularly anthocyanin mono- and diglucosides, remained stable, thereby preserving their potential biological activity within the intestinal environment. In the dynamic SHIME^®^ system, pomegranate juice administration modulated colonic metabolism and microbial composition over time and across the different colon compartments. Indeed, following an initial perturbation, total SCFA production increased, particularly in the DC, with a marked rise in butyrate levels. These findings suggest that the gut health effects of pomegranate juice may be mediated not only by the direct activity of its polyphenols, but also by their biotransformation by the gut microbiota, leading to microbial shifts and metabolite production along distinct colonic regions. Gut microbiota analysis revealed compartment-specific shifts, including an increase in beneficial taxa such as *Akkermansia*, *Bifidobacterium*, and butyrate-producing genera (e.g., *Enterocloster*), especially in the TC and DC. These findings suggest that pomegranate juice may exert prebiotic-like effects by modulating gut microbial communities toward a more beneficial profile and enhancing SCFA production. Overall, the combined antioxidant properties and microbiota-modulating capacity of Wonderful pomegranate juice support its potential role as a functional food for promoting gut health.

## Figures and Tables

**Table 1:** Production parameters of pomegranate fruits, cv. Wonderful, and polyphenolic composition and antioxidant activity of pomegranate juice (PJ). Values are expressed as mean ± SD (n = 3).

Productive Parameters		Polyphenol Composition of PJ		Antioxidant Activity of PJ	
Whole fruit (g)	554.4 ± 92.4	TPC (mg GAE/L)	2329.8 ± 10.1	DPPH IC50 (mg/L)	41.7 ± 3.3
Peels (g)	302 ± 36	TAC (mg C3GE/L)	602.6 ± 2.6	DPPH (µmol TE/mL)	12 ± 0.6
Arils (g)	252 ± 58.1	TFC (mg CE/L)	242.6 ± 3.1	ABTS (µmol TE/mL)	13.3 ± 4.6
Kernels (g)	67.5 ± 3.6			FRAP (µmol TE/mL)	19.7 ± 0.9
Juice (mL)	182.7 ± 55.3				

TPC: total phenolic content; TAC: total anthocyanin content; TFC: total flavonoid content; GAE: gallic acid equivalents; C3GE: cianidin-3-glucoside equivalents; CE: catechin equivalents; TE: Trolox equivalents.

**Table 2:** Polyphenol characterization of pomegranate juice by HPLC-DAD analysis.

Polyphenols	µg/mL
delphinidin 3,5-diglucoside	152 ± 5.31
cyanidin 3,5-diglucoside	1505.65 ± 18.21
delphinidin 3-O-glucoside	398.87 ± 6.54
cyanidin 3-O-glucoside	217.44 ± 2.01
pelargonidin 3-O-glucoside	11.96 ± 0.93
cyanidin glycoside	5.99 ± 0.11
ellagic acid derivative	12.89 ± 1.05
myricetin 3-O-glucoside	1.55 ± 0.09
ellagic acid	6.72 ± 0.28
total polyphenols identified	2312.07 ± 34.53

## Data Availability

The original contributions presented in this study are included in the article/Supplementary Materials. Further inquiries can be directed at the corresponding author.

## Supplementary Materials

The following supporting information can be downloaded at https://www.mdpi.com/article/10.3390/antiox15081047/s1: Figure S1: Millimolar concentration of minor SCFAs, in the different colon regions (ascending, transverse and descending), at 0, 1, 4, 7 days of pomegranate juice administration; Table S1: Concentrations of short-chain fatty acids (SCFAs) determined by GC-FID in the ascending, transverse, and descending colon, following pomegranate juice administration at 0, 1, 4, and 7 days. Values are expressed as mean ± SD. Different letters within rows indicate significant differences (*p* < 0.05) according to one-way ANOVA followed by Fisher’s LSD test. Figure S2: Alpha diversity (Shannon index) of the fecal microbiota in the colonic segment supplemented with pomegranate juice. AC = ascending, TC = transverse, DC = descending colon; Figure S3: Principal component analysis (PCoA) based on Bray–Curtis dissimilarity representing the beta diversity index of the fecal microbiota in the ascending colonic segment (AC) supplemented with pomegranate juice; Figure S4: Principal component analysis (PCoA) based on Bray–Curtis dissimilarity representing the beta diversity index of the fecal microbiota in the transverse colonic segment (TC) supplemented with pomegranate juice; Figure S5: Principal component analysis (PCoA) based on Bray–Curtis dissimilarity representing the beta diversity index of the fecal microbiota in the descending colonic segment (DC) supplemented with pomegranate juice.

## Abbreviations

The following abbreviations are used in this manuscript:

ABTS	2,2′-azino-bis (3-ethylbenzothiazoline-6-sulfonic acid) di-ammonium salt
AC	ascending colon
AcOH	glacial acetic
ASVs	amplicon sequence variants
C 3,5-diglu	cyanidin 3,5-diglucoside
C 3-glu	cyanidin 3-O-glucoside
C gly	cyanidin glycoside
C3GE	cyaniding 3-glucoside equivalent
D 3,5-diglu	delphinidin 3,5-diglucoside
D 3-glu	delphinidin 3-O-glucoside
DC	descending colon
DF	dilution factor
DPPH	1,1-Diphenyl-2-picryl-hydrazyl
DW	dry weight
EA	ellagic acid
EA der	ellagic acid derivative
EtOAc	ethyl acetate
F/B	Firmicutes/Bacteroidota
FRAP	ferric reducing antioxidant power
GAE	gallic acid equivalent
GC-FID	gas chromatography with flame ionization detection
H2SO4	sulphuric acid
HPLC-DAD	high-performance liquid chromatography with diode-array detection
IBD	inflammatory bowel disorders
LDA	linear discriminant analysis
LEfSe	linear discriminant analysis effect size
M 3-glu	myricetin 3-O-glucoside
MeOH	methanol
MW	molecular weight
NCBI SRA	short read archive at National Center for Biotechnology Information
P 3-glu	pelargonidin 3-O-glucoside
PJ	pomegranate juice
SHIME^®^	Simulator of the Human Intestinal Microbial Ecosystem
SI	small intestine
TAC	total anthocyanin content
TC	transverse colon
TE	trolox equivalents
TFC	total flavonoid content
TPC	total phenolic content
TPTZ	2,4,6-Tris (2-pyridyl)-s-triazine
