# Supplementary material for: A SHIME®-Based In Vitro Study to Simulate Human Gut Microbiota Modulation by Polyphenols from Pomegranate Juice
Source: Antioxidants (Basel). 2026 Aug 21;15(8):1047. doi: 10.3390/antiox15081047 (PMC13509527; doi:10.3390/antiox15081047)
Supplement: Supplementary file 1 [file antioxidants-15-01047-s001.zip › antioxidants-4427262-supplementary.pdf]

Supplementary Materials

Article

A SHIME®-Based *In Vitro* Study to Simulate Human Gut Microbiota Modulation by Polyphenols from Pomegranate Juice

Angelica Bruno, Massimo Ferrara *, Vito Linsalata, Angela Cardinali and Isabella D'Antuono *

Institute of Sciences of Food Production, National Research Council (CNR-ISPA), Via G. Amendola 122/O, 70126 Bari, Italy; angelicabruno@cnr.it (A.B.); vito.linsalata@cnr.it (V.L.); angela.cardinali@cnr.it (A.C.)

* Correspondence: massimo.ferrara@cnr.it (M.F.); isabella.dantuono@cnr.it (I.D.)

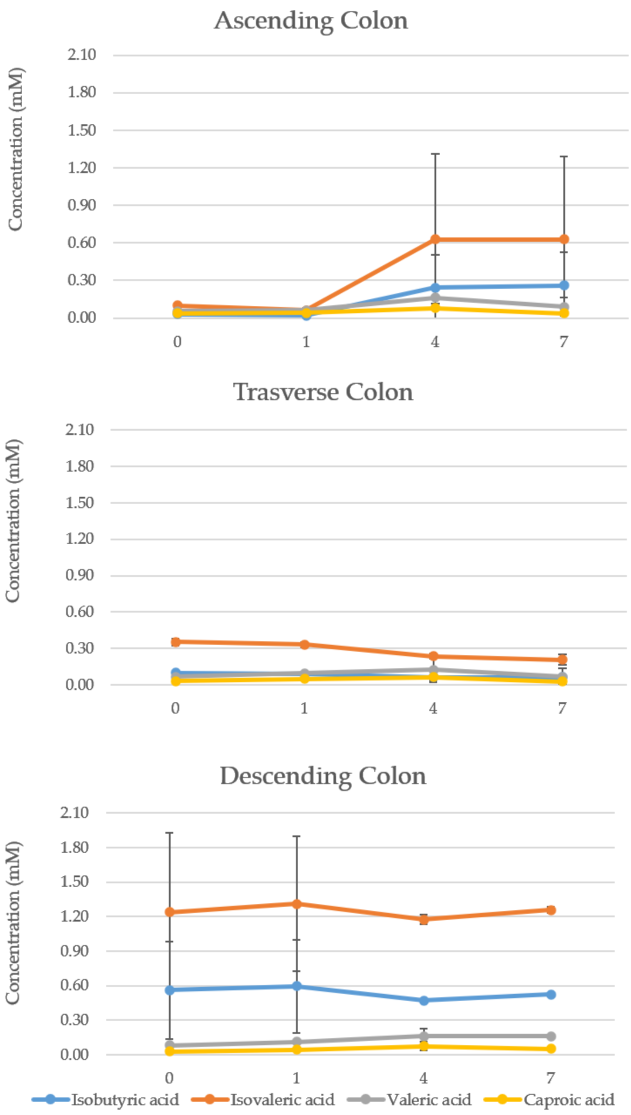

Figure S1. Millimolar concentration of minor SCFAs, in the different colon regions (ascending, transverse and descending), at 0, 1, 4, 7 days of pomegranate juice administration. Data are presented as the means ± SD.

Table S1. Concentrations of short-chain fatty acids (SCFAs) determined by GC-FID in the ascending, transverse, and descending colon, following pomegranate juice administration at 0, 1, 4, and 7 days. Values are expressed as mean ± SD. Different letters within rows indicate significant differences ($p < 0.05$) according to one-way ANOVA followed by Fisher's LSD test.

SCFAs	Day 0	Day 1	Day 4	Day 7
Ascending Colon				
Acetic acid	60.33±5.41 [b]	47.70±0.27 [c]	61.81±5.24 [a]	58.50±2.19 [a]
Propionic acid	5.32±0.34 [b]	4.99±0.33 [b]	7.91±2.45 [a]	9.76±2.21 [a]
Isobutyric acid	0.03±0.0	0.02±0.0	0.24±0.26	0.26±0.27
Butyric acid	8.90±0.72	6.47±1.50	8.96±5.73	6.77±5.59
Isovaleric acid	0.10±0.01	0.06±0.01	0.63±0.68	0.63±0.66
Valeric acid	0.06±0.0 [b]	0.06±0.02 [c]	0.16±0.07 [a]	0.09±0.07 [ab]
Caproic acid	0.04±0.0 [c]	0.04±0.01 [c]	0.08±0.03 [a]	0.04±0.01 [b]
TOT	74.78±5.79 [a]	59.34±1.60 [a]	79.79±14.22 [b]	76.04±10.80 [a]
Transverse Colon				
Acetic acid	64.31±1.29 [b]	51.02±1.42 [c]	68.33±1.24 [a]	67.78±2.64 [a]
Propionic acid	8.30±0.14 [b]	8.56±0.05 [b]	8.81±0.42 [b]	10.37±1.19 [a]
Isobutyric acid	0.10±0.01 [a]	0.09±0.01 [a]	0.07±0.0 [b]	0.06±0.01 [b]
Butyric acid	10.58±0.80	12.06±0.19	8.54±3.70	6.22±4.21
Isovaleric acid	0.35±0.03 [a]	0.33±0.02 [a]	0.24±0.02 [b]	0.21±0.06 [b]
Valeric acid	0.07±0.01	0.09±0.01	0.13±0.09	0.07±0.05
Caproic acid	0.03±0.01	0.05±0.0	0.06±0.05	0.03±0.01
TOT	83.74±2.15 [a]	72.21±1.52 [c]	86.18±3.04 [a]	84.74±5.23 [b]
Descending Colon				
Acetic acid	65.67±0.99 [b]	52.45±2.49 [c]	72.45±1.48 [a]	72.9±0.37 [a]
Propionic acid	9.28±0.16 [c]	9.93±0.74 [b]	10.06±0.25 [b]	12.39±0.12 [a]
Isobutyric acid	0.56±0.43	0.59±0.40	0.47±0.02	0.52±0.01
Butyric acid	10.88±0.66 [b]	12.39±0.91 [a]	12.74±1.17[a]	12.33±0.15 [a]
Isovaleric acid	1.24±0.69	1.31±0.58	1.17±0.04	1.26±0.02
Valeric acid	0.08±0.01 [b]	0.11±0.01 [ab]	0.16±0.07[a]	0.16±0.00 [a]
Caproic acid	0.03±0.01 [c]	0.04±0.01 [b]	0.07±0.04 [a]	0.05±0.00 [a]
TOT	87.73±2.90 [b]	76.82±3.62 [c]	97.13±2.33 [a]	99.62±0.67 [a]

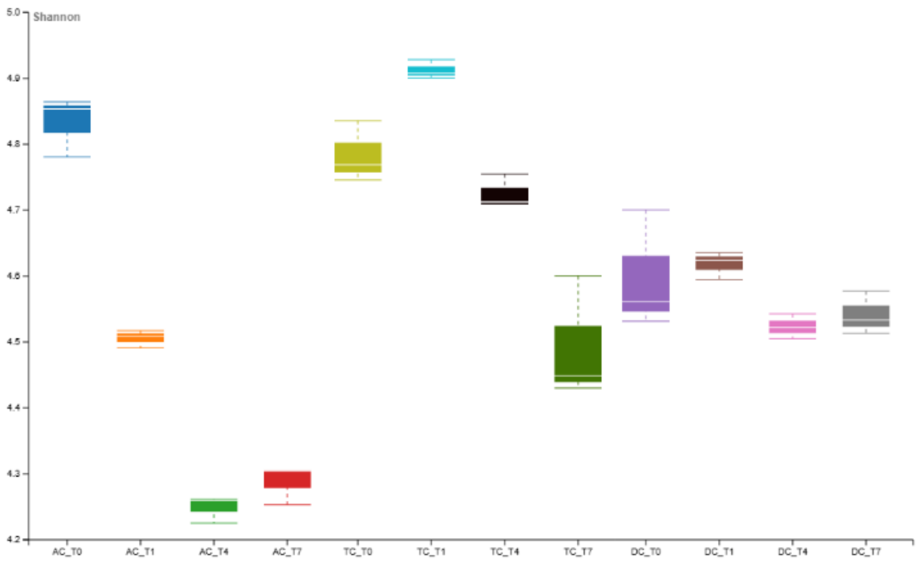

Figure S2. Alpha diversity (Shannon index) of the fecal microbiota in the colonic segment supplemented with pomegranate juice. AC=ascending, TC= transverse, DC=descending colon.

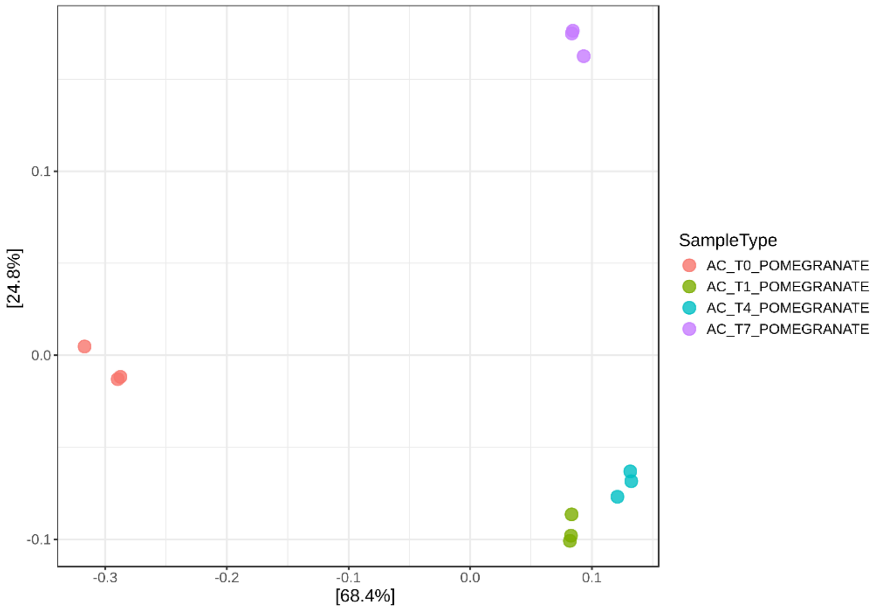

Figure S3. Principal component analysis (PCoA) based on Bray–Curtis dissimilarity representing the beta diversity index of the fecal microbiota in the ascending colonic segment (AC) supplemented with pomegranate juice.

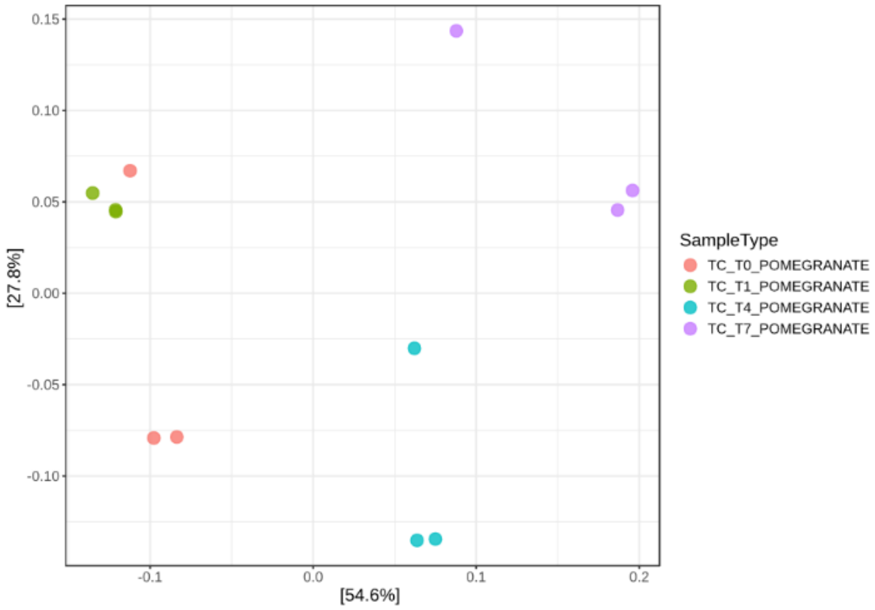

Figure S4. Principal component analysis (PCoA) based on Bray–Curtis dissimilarity representing the beta diversity index of the fecal microbiota in the transverse colonic segment (TC) supplemented with pomegranate juice.

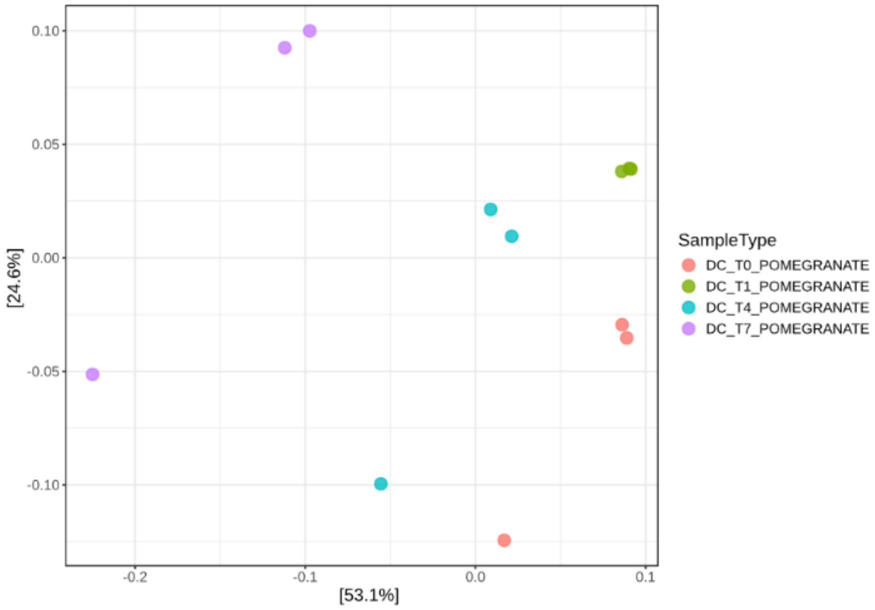

Figure S5. Principal component analysis (PCoA) based on Bray–Curtis dissimilarity representing the beta diversity index of the fecal microbiota in the descending colonic segment (DC) supplemented with pomegranate juice.
